# Interhypothalamic Adhesion in a 9-Month-Old Male with Cleft Palate

**DOI:** 10.1155/2013/197415

**Published:** 2013-12-04

**Authors:** Matthew T. Whitehead, Jacqueline D. S. Angel

**Affiliations:** ^1^Department of Radiology, University of Tennessee Health Science Center, Memphis, TN 38163, USA; ^2^Department of Radiology, Le Bonheur Neuroscience Institute, Le Bonheur Children's Hospital, 50 North Dunlap Street G216, Memphis, TN 38103, USA; ^3^The University of Tennessee Health Science Center, Graduate Medical Education, 910 Madison Avenue, Suite 1031, Memphis, TN 38163, USA

## Abstract

A 9-month-old male infant with multiple congenital anomalies including cleft lip and palate was referred to us for a brain MR to exclude additional intracranial abnormalities. Imaging revealed an interhypothalamic adhesion, which we present as a possible forme fruste of holoprosencephaly.

## 1. Introduction

Holoprosencephaly (HPE) represents a spectrum of disorders in which the prosencephalon, to varying degrees, fails to cleave into bilateral structures during embryologic development [1–4]. Though considered a disorder of telencephalic cleavage, it can also involve the diencephalon, which forms the thalamus and epithalamus [4]. The hypothalamus, now considered a derivative of the secondary prosencephalon, has been shown to be universally unionized in holoprosencephaly even in the mildest of forms [5]. HPE has been divided into three main types along its spectrum, in decreasing severity, as alobar, semilobar, and lobar. Other variants include syntelencephaly, solitary median maxillary central incisor syndrome, and congenital nasal pyriform aperture stenosis [1]. With the exception of syntelencephaly, the mildest forms of holoprosencephaly show only partial forebrain and diencephalic union.

We present a case of a 9-month-old male status after cleft palate repair, referred to us to evaluate additional intracranial congenital anomalies. Brain MR revealed an interhypothalamic adhesion in addition to falx cerebri hypoplasia, hip- pocampal malrotation, and equivocal optic tract hypoplasia.

## 2. Case

A 9-month-old male infant with multiple congenital anomalies, including a history of repaired unilateral complete cleft palate and lip, released club foot, and bilateral undescended testes, presented to our imaging service to exclude additional intracranial congenital abnormalities. The patient had previously undergone an extensive diagnostic workup that revealed a normal male genetic karyotype. As of this case writeup, the workup had not rendered a specific unifying diagnosis.

A brain MRI was requested to search for congenital intracranial structural midline abnormalities. The examination was performed on a 1.5T MR (Signa HDxt Optima edition, General Electric, Milwaukee, WI, USA). Prescribed pulse sequences included sagittal T1WI fast spoiled gradient echo Brain Volume imaging (FSPGR BRAVO), axial T1 fluid attenuation inversion recovery (FLAIR), axial T2WI, axial T2 fluid attenuation inversion recovery (FLAIR), and coronal short tau inversion recovery (STIR). In addition, thin section coronal and axial T1WI FSPGR and coronal fast imaging employing steady-state acquisition cycled phases (FIESTA-C) were performed through the sella turcica and hypothalamus. Finally, postcontrast whole brain axial T1WI was acquired along with coronal and sagittal T1W FSPGR images through the sella turcica and hypothalamus after intravenous administration of 2 mL gadopentate dimeglumine (Magnevist, Bayer HealthCare Pharmaceuticals, Berlin, Germany).

**Figure 1:**
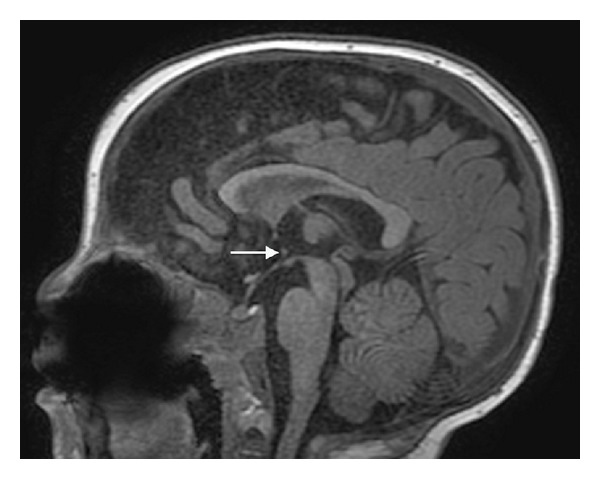
Sagittal FSPGR BRAVO T1WI (repetition time msec/echo time msec/inversion time msec, 10/4/450) showing a nodular parenchymal band in the third ventricle at the level of the hypothalamus between the anterior commissure and mammillary body (arrow). The pituitary infundibulum, adenohypophysis, and neurohypophysis are normal. Note metallic susceptibility artifact in the roof of the oral cavity from prior cleft palate repair.

**Figure 2:**
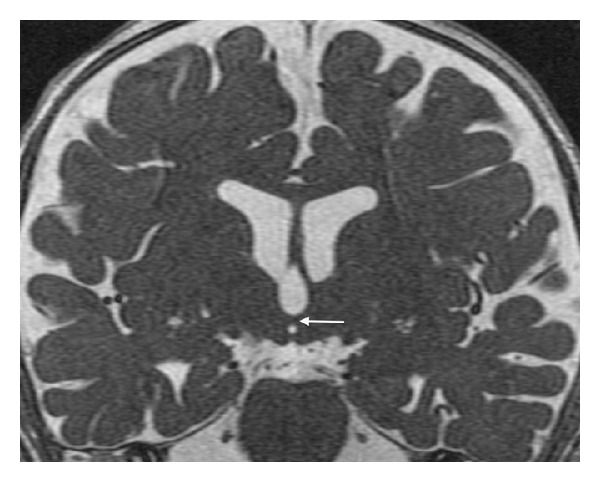
Coronal FIESTA-C (repetition time msec/echo time, 10/4) showing a horizontal interhypothalamic parenchymal band crossing the third ventricle (arrow).

**Figure 3:**
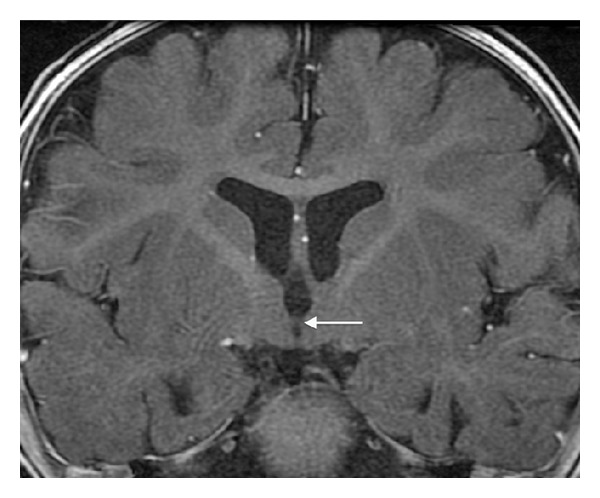
Postcontrast coronal FSPGR T1WI (repetition time msec/echo time msec/inversion time msec, 19/9/375) depicting a parenchymal band that is isointense to gray matter crossing the third ventricle and connecting the medial hypothalami (arrow).

**Figure 4:**
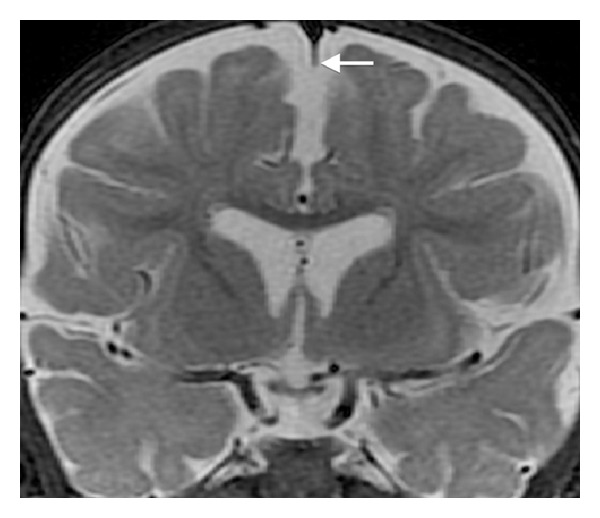
Coronal STIR (repetition time msec/echo time msec/inversion time msec, 4217/83/150) image demonstrating partial deficiency of the falx cerebri (arrow). Note the normal septum pellucidum.

**Figure 5:**
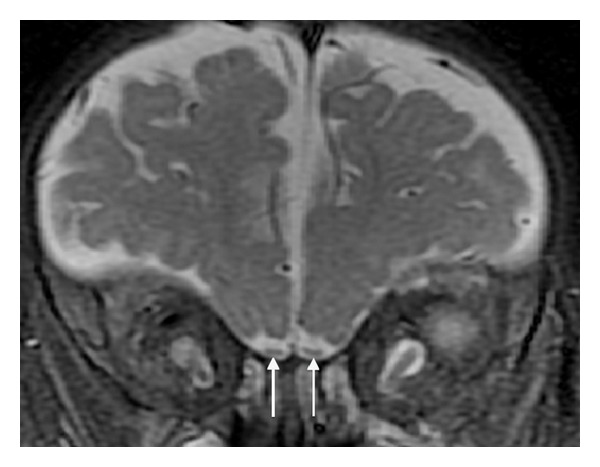
Coronal STIR (repetition time msec/echo time msec/inversion time msec, 4217/83/150) image demonstrating equivocal olfactory tract volume loss (arrows).

**Figure 6:**
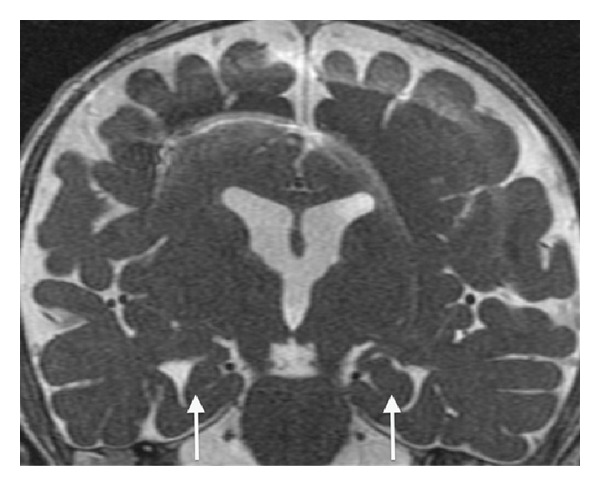
Coronal FIESTA-C (repetition time msec/echo time, 10/4) demonstrating malrotation of the hippocampal heads (arrows).

Midline sagittal T1WI reveals an unusual small nodular structure in the inferior aspect of the third ventricle at the level of the posterior hypothalamus (Figure 1). Coronal FIESTA-C image shows that the nodule represents an abnormal horizontal band of parenchymal tissue traversing the third ventricle and connecting the medial aspects of the posterior hypothalami (Figure 2). The unusual parenchymal tissue is isointense to gray matter with T1 (Figure 3) and T2 prolongation. The falx cerebri is partially deficient (Figure 4). Although present, there is equivocal symmetric olfactory tract volume loss (approximate maximal diameter: 3 mm transverse × 1 mm craniocaudal dimension); the olfactory sulci are normally formed (Figure 5). The transverse axes of the hippocampal heads are malrotated with hippocampal infolding angles (HIA) of roughly 45 degrees bilaterally (Figure 6); HIA was measured in accordance with previous techniques described in the literature [6, 7]. Diencephalic, pituitary, and forebrain structures are otherwise normal. The septum pellucidum is present (Figure 4).

## 3. Discussion

In normal neurological development, primary neurulation results in the cranial neural tube dividing into three primary brain vesicles: prosencephalon, mesencephalon, and rhombencephalon. The prosencephalon (forebrain) then undergoes cleavage and midline development, giving rise to two secondary vesicles, the telencephalon (cerebral hemispheres, basal ganglia, and hypothalamus) and the diencephalon (thalamus and epithalamus), and, ultimately, the forebrain structures [2, 4].

Holoprosencephaly (HPE) represents a spectrum of disorders encompassing a variable degree of nonseparation of the prosencephalon structures. In humans, it is the most common developmental defect of the forebrain and midface. The range of classical HPE includes three subtypes, from most severe to least severe: alobar, semilobar, and lobar [2, 4]. Additional variants exist, including syntelencephaly/middle interhemispheric variant (MIH), solitary median maxillary central incisor syndrome, and congenital nasal pyriform aperture stenosis [1]. With the exception of syntelencephaly, the mildest forms of HPE show only partial forebrain and diencephalic union. However, the hypothalamus has been shown to be universally unionized in HPE, even in the mildest of forms [5]. In addition to the neurological manifestations of HPE, many individuals suffer from other midline defects, such as anophthalmia, cyclopia, proboscis, cleft lip, cleft palate, absent septum pellucidum, and/or absent corpus callosum [8].

Complex gene and environmental interactions can lead to the HPE phenotypic gradations, but many of the mechanisms involved in HPE are not fully understood [8, 9]. A multitude of genes, including SHH, BMP, ZIC2, SIX3, DISP1, FOXH1, GLI2, PTCH1, TGIF, and NODAL, and karyotype variations have been linked to HPE [8, 10–12]. Genetic dysfunctions of SHH (responsible for ventral patterning) and BMP (involved in dorsal patterning), either directly or indirectly, tend to result in holoprosencephaly. These genes work in opposition along the length of the neural axis; diminished or enhanced activity of either of these genes can lead to HPE [11]. Classical HPE disease spectrum (lobar, semilobar, and alobar) is associated with decreased SHH activity or increased BMP activity. MIH HPE results from increased SHH activity or decreased BMP activity.

Brain development is the greatest in lobar HPE as compared to the other classical subtypes [1]. In this form, the individual has separation of the majority of the cerebral hemispheres with possible union of the ventral frontal lobes [12]. The third and lateral ventricles, as well as the interhemispheric fissure and falx, tend to be present and relatively well formed. The olfactory tracts and bulbs and/or the corpus callosum can be absent, hypoplastic, or normal [13]. The hippocampi may be more vertically oriented, and the septum pellucidum can be absent [6, 13]. Other findings may include a cleft lip, flat nose, and ocular hypotelorism but with an otherwise somewhat normal face. Many other midline craniofacial or neurological anomalies may also be present.

We discovered a gray matter band connecting the medial margins of the posterior hypothalami across the third ventricle in our patient. Simon et al. described some degree of hypothalamic union in all HPE patients in their cohort [5]. Therefore, we believe that our patient exhibits a forme fruste holoprosencephaly despite having a normal septum pellucidum. The presence of additional abnormalities that have been previously described in HPE including cleft lip/cleft palate, falx cerebri hypoplasia, and malrotation of the hippocampi lends further credence to the diagnosis. Moreover, equivocal symmetric hypoplasia of the optic tracts in our patient is an additional finding that can be seen in lobar HPE.

## 4. Conclusion

Mild intracranial anomalies including an interhypothalamic adhesion, falx hypoplasia, hippocampal malrotation, and possible olfactory tract hypoplasia are present in this infant with a history of cleft lip and palate. As HPE exists along a phenotypic spectrum of varying anomalies, we believe that our case of interhypothalamic adhesion exemplifies an attenuated form of lobar HPE.
